# Noninvasive genetic population survey of snow leopards (*Panthera uncia*) in Kangchenjunga conservation area, Shey Phoksundo National Park and surrounding buffer zones of Nepal

**DOI:** 10.1186/1756-0500-4-516

**Published:** 2011-11-28

**Authors:** Dibesh B Karmacharya, Kamal Thapa, Rinjan Shrestha, Maheshwar Dhakal, Jan E Janecka

**Affiliations:** 1 Center for Molecular Dynamics Nepal, Swaraj Sadan 5th Floor, Thapathali-11, Kathmandu, Nepal; 2 World Wildlife Fund Inc., Nepal Programme Office, PO Box 7660, Baluwatar, Kathmandu, Nepal; 3 Department of National Parks and Wildlife Conservation, Ministry of Forests and Soil Conservation, Kathmandu, Nepal; 4 Department of Veterinary Integrative Biosciences, College of Veterinary Medicine and Biomedical Sciences, Texas A&M University, College Station, Texas, USA

## Abstract

**Background:**

The endangered snow leopard is found throughout major mountain ranges of Central Asia, including the remote Himalayas. However, because of their elusive behavior, sparse distribution, and poor access to their habitat, there is a lack of reliable information on their population status and demography, particularly in Nepal. Therefore, we utilized noninvasive genetic techniques to conduct a preliminary snow leopard survey in two protected areas of Nepal.

**Results:**

A total of 71 putative snow leopard scats were collected and analyzed from two different areas; Shey Phoksundo National Park (SPNP) in the west and Kangchanjunga Conservation Area (KCA) in the east. Nineteen (27%) scats were genetically identified as snow leopards, and 10 (53%) of these were successfully genotyped at 6 microsatellite loci. Two samples showed identical genotype profiles indicating a total of 9 individual snow leopards. Four individual snow leopards were identified in SPNP (1 male and 3 females) and five (2 males and 3 females) in KCA.

**Conclusions:**

We were able to confirm the occurrence of snow leopards in both study areas and determine the minimum number present. This information can be used to design more in-depth population surveys that will enable estimation of snow leopard population abundance at these sites.

## Background

**Figure 1:**
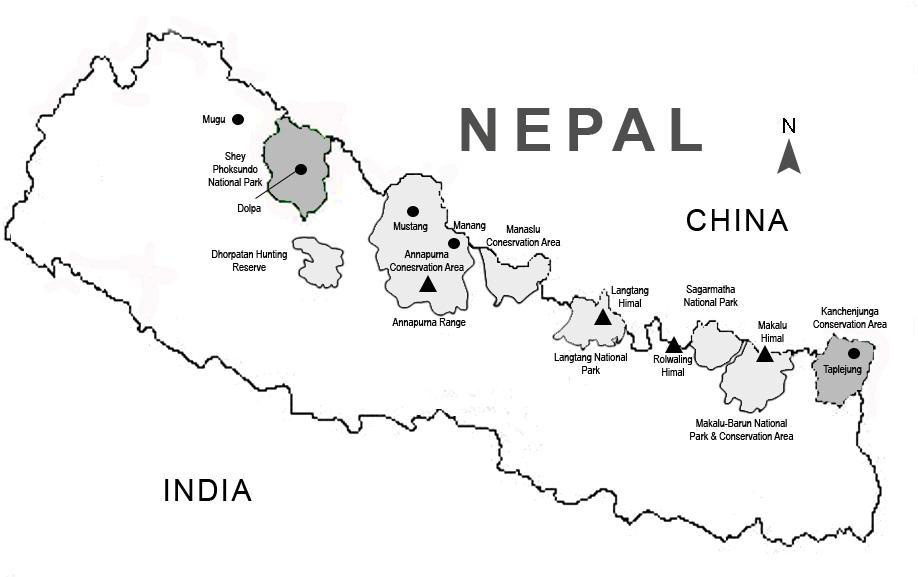
**Map of the study sites**. The snow leopard study sites (highlighted) - Shey Phoksundo National Park (SPNP) in the west and Kanchanjunga Conservation Area (KCA) in the east of Nepal. Annapurna Conservation Area, Rolwaling Himal and Sagarmatha National Park are also prime habitat for snow leopards. There are estimated 350-500 snow leopards in Nepal.

The snow leopard (*Panthera uncia*) is among the most elusive felids. It is widely distributed throughout the alpine ecosystems of Central Asia, including mountains of the Himalaya, Karakoram, Pamir, Tien Shan, and Altai. Its range broadly covers 12 countries; Afghanistan, Bhutan, China, India, Kazakhstan, Kyrgyzstan, Mongolia, Nepal, Pakistan, Russia, Tajikistan and Uzbekistan [1-3]. However, locally snow leopard distribution is often fragmented and patchy (Figure 1). A habitat suitability index model predicts an estimated 350-500 snow leopards distributed across the 30, 000 km^2 ^of Nepal's northern frontier [3,4]. Despite being a conservation priority in Nepal and their protection under Schedule 1 of National Parks and Wildlife Conservation Act 1973, their numbers are believed to be declining [4]. The long-term viability of snow leopards is threatened by anthropogenic factors such as reduction of prey populations, poaching, and conflict with locals [5]. There is active illicit trans-border trafficking of wildlife parts, including those from snow leopards, between India, Nepal and China/Tibet [5]. Global climate change could also potentially affect the distribution of snow leopards in Nepal by reducing available habitat. However, before an effective conservation strategy for snow leopards can be designed and implemented in Nepal it is crucial to obtain quantitative information on their distribution and abundance.

The data available on the status of snow leopards in Nepal is limited and inadequate largely because of the logistical challenges of studying this elusive and solitary species, which dwells in rugged, inaccessible habitat [6,7]. Most research to date relied on signs surveys (e.g. pugmarks, scrapes and scats), interviews with local inhabitants, and camera trapping [2,7-10]. These approaches have several disadvantages. Sign-based surveys and interviews with locals yield only qualitative information, which also may not always be credible. Camera-trapping in remote snow leopard habitat is difficult and requires a large amount of field time (> 8 weeks) resulting in high costs [6]. Hence, additional methods that yield quantitative data are essential for the effective monitoring of snow leopards [11,12].

Noninvasive genetic analysis has become widely used in wildlife research [13]. This technique avoids potential adverse effects from direct contact and is favored when dealing with rare and endangered species [12]. The success of previous noninvasive genetic carnivore studies suggested that this approach would be useful for monitoring snow leopards [14]. Indeed it was found to be particularly effective as snow leopards use predictable paths, frequently mark by scraping, and deposit scat and urine in distinctive places, thereby facilitating non-invasive sampling [11,13]. Molecular markers have been recently designed to identify species, sex and individuals from DNA extracted from snow leopard scats [11]. Additional available molecular markers can provide insight into landscape connectivity, population structure, migration rates, effective population size, recent bottlenecks and kinship [15,16].

Noninvasive methods using scat have been successfully employed to detect low density, wide-ranging and elusive species [11,17]. A recent study on snow leopard in Mongolia showed that the technique was very effective for detecting individuals and could be used for population estimation [11,12]. The ability to detect snow leopards via collection of scats and generate population abundance estimates was found to be more cost and time effective than camera trapping [12]. In this study, we aimed to determine the presence of snow leopards by analyzing putative scat samples collected from two sites in Nepal and obtain a preliminary estimate of abundance.

## Results

We collected a total of 23 samples from Shey Phoksundo National Park (SPNP) and 48 samples from Kangchanjunga Conservation Area (KCA) between 2006 and 2009. Forty-nine of the 71 (69%) scats were positive in the carnivore-specific PCR assay. Out of these 49 samples, 19 (39%) were identified as originating from a snow leopard using the species-specific PCR assay: 10 of these were from KCA (8 from Ghunsa and 2 from Yagma regions) and 9 were from SPNP (4 from Shey and 5 from Dho regions). There was 100% congruence in the results of the two PCR assays; all of the identified snow leopard scats were also determined to be carnivores (Table 1).

**Table 1:** Overall result of Species and Carnivore specific PCR identification:

	Carnivore PCR Positive sample	Carnivore PCR Negative sample	TOTAL(N)
Species PCR Positive samples	N = 19	N = 0	Total Species ID PCR positive = 19
Species PCR Negative samples	N = 30	N = 22	Total Species ID negative = 52
Total (N)	N = 49	N = 22	Total No. of samples = 71

All 19 snow leopard positive PCR samples were also positive for Carnivore specific PCR. There were 30 samples that were positive for carnivore PCR but negative for snow leopard PCR. For both species and carnivore specific PCR, there were 22 samples that did not yielded any amplification.

Ten of the 19 (53%) snow leopard scats were genotyped successfully across 6 microsatellites. As there was no other snow leopard sample available from these populations, we had to estimate the probability of identity (PID) from the allele frequencies among the unique composite genotypes observed in our sample. The P_ID-unrelated _and P_ID-sibling _was 0.00014 and 0.0182, respectively. Overall expected H_e _was 0.579 and H_o _was 0.631 (Table 2). There was total of 9 (47%) individuals observed: 4 in SPNP (3 from Shey and 1 from Dho) and 5 from KCA (3 from Ghunsa and 2 from Yagma) (Table 3). Two scats that were collected at sites approximately 4.0 km apart had an identical composite genotype and were therefore from the same individual.

**Table 2:** Genetic diversity on 6 selected microsatellite loci.

Locus	N	Na	Ne	Ho	He	UHe
**PUN124**	8	6	3.2	0.750	0.688	0.733
**PUN229**	8	4	3.5	0.875	0.711	0.758
**PUN935**	7	4	3.3	0.714	0.694	0.747
**PUN1157**	6	3	2.6	0.667	0.611	0.667
**PUN132**	9	4	2.3	0.556	0.574	0.608
**PUN894**	9	2	1.2	0.222	0.198	0.209

**Table 3:** Individual identification through genotyping: Six microsatellite loci (PUN124, PUN 229, PUN935, PUN1157, PUN132 and PUN894) were selected for individual identification.

SITE	YEAR	**IND**.	PUN124		PUN229		PUN935		PUN1157		PUN132		PUN894	
***Shey Phoksundo National Park***														
**Shey**	2008	1	90	96	106	112	121	121	101/ 103	103	118	118	110	110
**Shey**	2008	2	96	90	102	106	115	119	105	105	112	116	110	110
**Shey**	2008	2	96	90	102	106	115	119	105	105	112	116	110	110
**Shey**	2008	3	90	92	106	112	121	121	101	101	118	120	110	110
**Dho**	2008	4	94	96	102	106	119	121	101	105	118	118	110	110
***Kanchenjunga Conservation Area***														
**Yagma**	2009	5	96	98	106	110	115	119	103	105	112	118	110	110
**Gunsa**	2009	6	89	98	106	110	119	123	103	105	112	118	110	118
**Yagma**	2009	7	-	-	110	112	115	115/ 119	101/ 109	109	118	118	110	118
**Gunsa**	2009	8	96	96	108/ 110	110	115/ 119	119	105/ 101	105	112	112	110	110
**Gunsa**	2006	9	96	96	110	110	115	119	101	105	112	118	110	110

Ten samples, which were snow leopard positive on species PCR identification, successfully amplified across all 6 microsatellite loci. There were two samples from Shey area of SPNP that had complete match across all 6 microsatellite loci and were identified as samples from the same individual. A total of 9 snow leopards were identified from SPNP and KCA.

**Figure 2:**
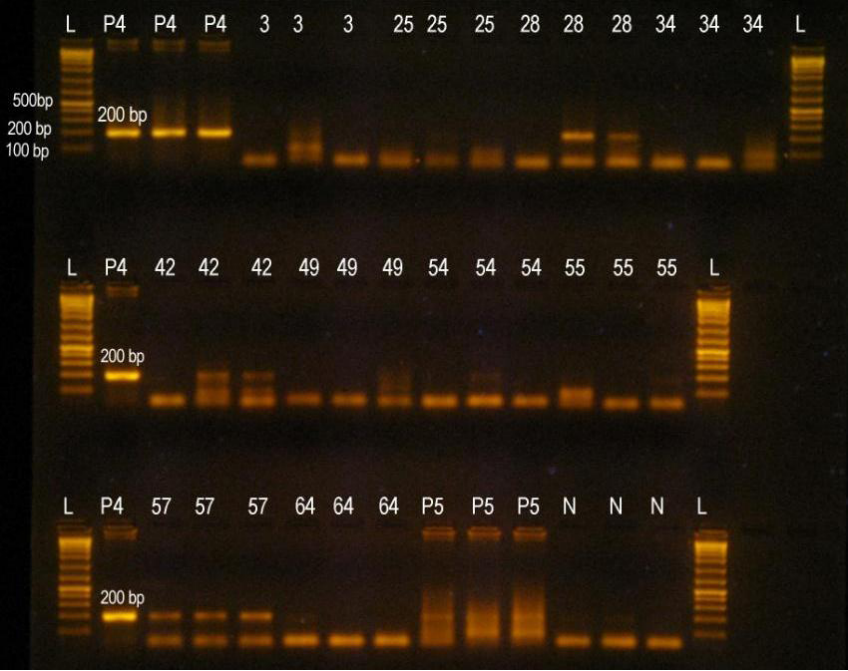
**Sex identification PCR result**. Gel electrophoresis image of Sex identification PCR. 200-bp PCR fragment was obtained amplifying portion of AMELY gene. L = 1 Kb Ladder. P4 = Known Male snow leopard sample. P5 = Known Female snow leopard sample. N = No template control. Three samples were identified as males and six as females (with two samples from same individual-Table 3).

The sex-identification PCR revealed 3 (33%) of the 9 individuals were male and the other 6 (67%) were female (Table 4, Figure 2). Among the 3 males identified, 1 was from Shey region of SPNP while the other 2 were from Yagma and Gunsa (all in KCA). And among the 6 individual females, 3 were from Shey and Dho regions of SPNP and the other 3 were from Ghunsa and Yagma regions of KCA (Table 4).

**Table 4:** Sex identification PCR results performed in genotyped successful samples.

S.N	SL	Location	Gender
**SPNP**			
**1**	49	Shey	Female
**2**	54	Shey	Female
**3**	55	Shey	Female
**4**	57	Shey	Male
**5**	64	Dho	Female
**KCA**			
**6**	42	Yagma	Male
**7**	34	Yagma	Female
**8**	28	Gunsa	Male
**9**	25	Gunsa	Female
**10**	3	Gunsa	Female

Altogether there were 10 samples that genotyped across all 6 microsatellite loci. Sample #54 and 55 had complete match on all the microsatellite loci, and hence were identified as samples coming from the same individual.

## Discussion

A total of 19 (27%) snow leopard scats were identified out of the 71 samples collected. A large proportion of the scat presumed to have come from snow leopards were either misidentified in the field (42%) or too degraded for genetic analysis (31%). Thirty of the scats were identified as carnivores but failed to amplify using the snow leopard PCR assay. These likely belonged to other common carnivores such as red fox (*Vulpes vulpes*), dog (*Canis familiaris*), wolf (*Canis lupus*), lynx (*Lynx lynx*), or other sympatric species. Mis-identication of carnivore scats in the field is a frequent problem in many studies focusing on felids [11,12,18,19]. Further genetic analysis, such as sequencing a portion the *cytochrome b *[11,19] is needed to identify the 30 unknown carnivore scats. Additionally, introduction of scat detection dogs trained to identify snow leopard scats in the field could minimize misidentification of scat in the field [20]. Since our primary objective was to determine the presence and abundance of snow leopards and we had limited resources, we designed a snow-leopard PCR assay to reduce the costs of screening scats.

There were 22 samples that for which there was no PCR amplification in either the snow leopard-specific PCR assay or the PCR using primers that were designed for carnivores. These scats could either be from species in which the primers did not work, or represent samples that did not yield DNA suitable for genetic analysis. Some of the samples were stored for more than 3 years under suboptimal conditions prior to analysis, likely contributing to the relatively low success rate we observed compared to other studies [11,12,19].

We had a much lower proportion of snow leopard scat among the samples collected (27%) than previous studies [11]. Much of this could be largely attributed to conditions under which samples were stored. The scats were collected by WWF-Nepal for a diet study rather than a non-invasive genetic population survey. The scats were stored in a tube with silica desiccant. When the samples were deposited at CMDN for genetic analysis, the indicator silica desiccant was saturated for moisture and therefore the material was not dry for a large amount of time. This appears to have lead to DNA degradation, fungal and bacterial growth on scat, and disintegration of the outer layer of scat, contributing to the difficulties observed during the genetic analysis.

We had a relatively low success with individual identification using microsatellites; out of the 19 snow leopard samples only half (10) were genotyped across all 6 microsatellite loci. The remaining 9 samples had genotyping error in the form of amplification failure and allele dropout. This also indicated that the DNA quality of these samples was poor. It is imperative that proper sample collection and storage protocols are followed so that accurate genetic data can be generated.

## Conclusions

Our noninvasive genetic population survey of snow leopards represents the first such work completed in Nepal by a local laboratory. In collaboration with foreign partners, we were able to perform species, sex, and individual identification from 2 - 3 year old scats collected by WWF-Nepal team. As most of these samples were collected for diet analysis, they were stored at room temperature without any desiccant until 2008, contributing to the low success rate we had with genetic analysis. Nonetheless, our discovery of 9 individual snow leopards occurring in two different protected areas in Nepal is very encouraging; this study has paved the way to carry out similar surveys in the near future.

Snow leopards have been previously observed across Nepal in its mountainous protected areas and our study confirms recent presence in two of these areas; KCA in the east and SPNP in the west. Meanwhile, the country's largest known snow leopard populations are believed to be in Dolpa, Mugu, Manang, Mustang, and Taplejung districts [5]. Snow leopards have been sighted in the northern region of the Annapurna range [3]. Ahlborn and Jackson [15] reported a density of at least 5-10 snow leopards per 100 km^2 ^in the remote, uninhabited Langu Valley of western Nepal. Snow leopard presence has been confirmed using traditional survey methods in various protected areas of Nepal [3,15,21]. Despite these efforts, there is a lack of quantitative estimates of population size in these areas. The majority of the current estimates are the result of qualitative Snow Leopard Information Management System (SLIMS)[2] type surveys, limited camera trapping, and extrapolation of information across suitable habitat.

Rigorous and systematic studies incorporating noninvasive genetics along with conventional methods of gathering information are needed to obtain reliable estimates of the existing snow leopard populations in Nepal. Currently, this country is experiencing rapid development and information on how human activities are affecting snow leopard population are needed to identify areas of high conservation priority and to assess the effectiveness of conservation actions. In addition, by expanding genetic-based studies to the landscape level, we can explore other important aspects including overall genetic diversity, population structure, dispersal patterns, and social structure, all of which will contribute to our understanding of snow leopard ecology in the central Himalaya region.

## Methods

### Sampling

Putative snow leopard scat samples were collected from SPNP and KCA of Nepal throughout all seasons with the permission from the Department of National Parks and Wildlife Conservation, Ministry of Forests and Soil Conservation, Government of Nepal, between 2006 to 2009 by field biologists of WWF-Nepal and trained locals (Figure 1). These scat samples were collected primarily for a dietary study of snow leopards. SLIMS and adopted Snow Leopard Monitoring Guideline [22] were used as field references when collecting putative snow leopard scats. Ridgelines, cliff bases and outcrops including livestock trails were searched for scat. Putative snow leopard scats were identified in the field based on the size, shape, odor, and other associated field signs (i.e., proximity to tracks, scrapes, and sprays). Samples were preserved in containers with silica desiccant at room temperature.

### Scat DNA Extraction

The scat samples were analyzed in the laboratory of the Center for Molecular Dynamics Nepal (CMDN). The peripheral layer of dried scat was scraped with sterile scissors and tweezers and approximately 200 mg was used for DNA extraction with the Qiagen QIAamp DNA Stool kit (Qiagen, Valencia, CA, USA) per manufacturer instruction.

### Carnivore Verification

Confirmation that scats were from a carnivore and had DNA suitable for downstream analysis was done by amplifying 148 bp region of mitochondrial *cytochrome b *gene using carnivore-specific primers:

CYTB-SCT-F' (5'-AAACTGCAGCCCCTCAGAATGATATTTGTCCTCA-3')

CYTB-SCT-R' (5'-TATTCTTTATCTGCCTATACATRCACG-3') [18]

A 25 μl PCR reaction was prepared containing 2.5 μl of 10 × Pfu Buffer, 2.5 μl of 25 mM MgCl_2_, 0.8 μl of 25 mM dNTPs, 0.25 μl of 10 μg/μl BSA, 0.10 μl of 5 U/μl Pfu *taq *enzyme (ShinGene Molecular Biotech Inc., Shanghai, China), 0.50 μl of each 20 μM primer, 16.35 μl of distilled water, 2.0 μl of the DNA extract. The PCR reaction were carried out at the following thermocycling condition: 96°C for 4 minutes followed by 40 cycles of each 95°C for 30 seconds, 60°C for 30 seconds and 72°C for 1 minute, with a final extension at 72°C for 5 minutes. The PCR products were visualized under ultraviolet light on a 2% agarose gel stained with ethidium bromide.

### Species Identification

**Figure 3:**
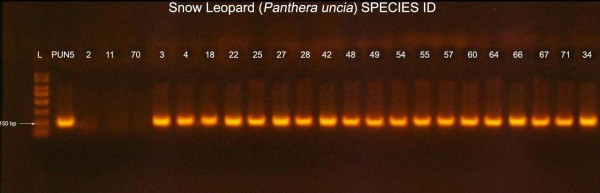
**Snow leopard species identification PCR result**. Gel electrophoresis image of snow leopard species identification PCR. The snow leopard specific target amplifed a 150-bp region of *cytochrome b *of mitochondrial DNA. L = 1 Kb Ladder; PUN5 = Known snow leopard Sample. Samples # 2, 11 & 70 are negative samples for snow leopard specific PCR. Rest of the samples are snow leopard specific PCR positive samples.

**Figure 4:**
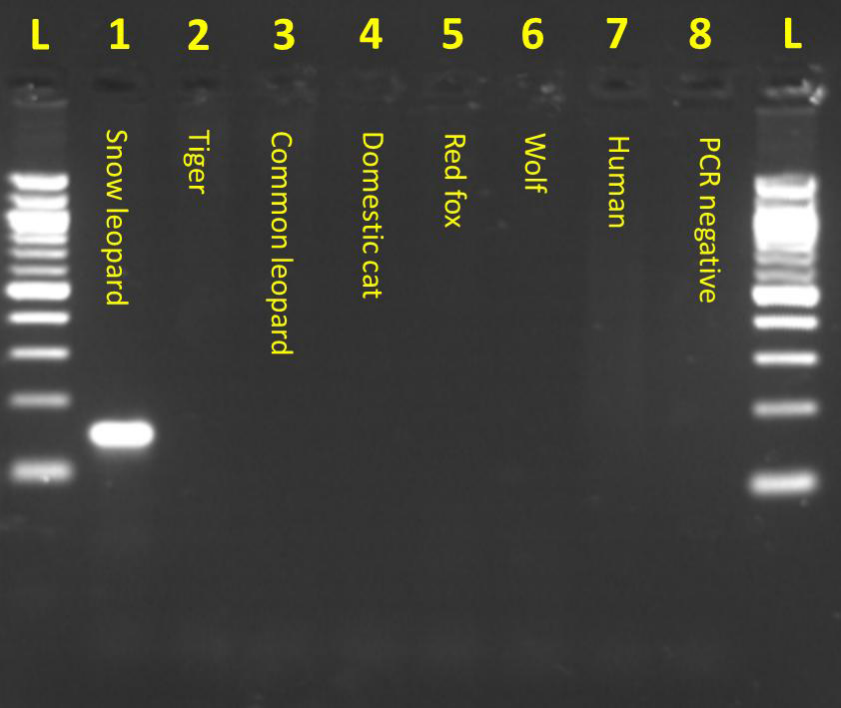
**Snow leopard species identification PCR assay specificity**. Snow leopard-specific PCR assay results showing amplification in only the snow leopard sample and complete lack of amplicons in other species assayed. The bottom two bands in the ladder are 100 bp and 200 bp, respectively.

Identification of snow leopard scats was carried out using a PCR assay developed at Texas A&M University using snow leopard-specific primers (Figure 3). To design the primers, available *cytochrome b *sequences were downloaded from GenBank and included snow leopard (NC_010638), tiger (EF551003, AF053048, AF053022, AF053050), leopard (EF551002, AY250072, AY250053), Eurasian lynx (LYXMTCYTB) AY928671, domestic cat (AB194817), wolf (AY598494S06), red fox (AY928669), sheep (AF010406), pica (AJ537415), and human (NC001807). The forward 20-bp primer were designed in an annealing site that had at least 2 mismatches at the 3'-end with any other species and the reverse primer was designed so that it had > 4-bp mismatch. The primers were tested at 1.5 mM MgCl_2 _and at annealing temperatures from 55°C to 65°C for amplification in snow leopard, tiger, leopard, domestic cat, wolf, red fox, and human (Figure 4). The primers did not amplify in any other species when annealing temperature was above 58°C, however, to be conservative we used an annealing temperature 60°C for testing scats.

CYTB-SCT-PUN-F' (5'-TGGCTGAATTATCCGATACC)

CYTB-SCT-PUN-R' (5'-AGCCATGACTGCGAGCAATA)

These primers amplify an approximately 150-bp region of *Cytochrome b *of mitochondrial DNA.

A 10 μl PCR reaction was prepared containing 1 μl of 10 × Pfu Buffer, 0.6 μl of 25 mM MgCl_2_, 0.32 μl of 25 mM dNTPs, 0.1 μl of 10 μg/μl BSA, 0.04 μl of 5 U/μl Pfu enzyme, 0.20 μl of each 20 μM primer, 6.04 μl of distilled water, and 1.7 μl of DNA extract. The PCR reaction was carried out using the following thermo-cycling condition: 96°C for 4 minutes followed by 40 cycles of each 95°C for 30 seconds, 60°C for 30 seconds and 72°C for 1 minute with a final extension at 72°C for 5 minutes. Each assay was done in triplicate and included a known snow leopard DNA positive control and a "no DNA template" negative control. The PCR products were visualized under ultraviolet light on a 2% agarose gel stained with ethidium bromide.

### Sex Identification

The sex identification of the verified snow leopards scat samples was carried out by testing for the presence of the Y chromosome using primers that amplified an intron of the AMELY gene (i.e., a gene only found on the Y). These primers were developed by Murphy et al [18] and it was previously verified they do not amplify in female felids [11,18].

AMELY-F' (5'-CCCAGCACACTCCTATTTGG-3')

AMELY-R' (5'-GGAATTTCAGCTGCAAAGGA-3')

A PCR reaction of total volume 10 μl was prepared containing 1 μl of 10 × Taq Polymerase Buffer, 0.8 μl of 25 mM MgCl2, 0.20 μl of 10 mM dNTPs, 0.1 μl of 10 μg/μl BSA, 0.05 μl of 5 U/μl Taq Polymerase enzyme, 0.24 μl of each primer, 5.87 μl of distilled water, and 1.5 μl of extracted undiluted DNA was added. The PCR reaction were performed using the following thermo-cycling condition: 95°C for 10 minutes; 94°C for 2 minutes followed by 50 cycles of each 94°C for 15 seconds, 55°C for 30 seconds and 72°C for 1 minute. Each sample was run in triplicate along with known snow leopard male positive control and snow leopard female negative controls. The PCR products were visualized under ultraviolet light on a 2% agarose gel stained with ethidium bromide. Male samples yielded an approximately 200 bp PCR fragment, while there was no PCR amplification on the female samples.

### Individual Identification

Six microsatellite were genotyped in the samples verified as originating from snow leopards (PUN124, PUN132, PUN229, PUN894, PUN935 and PUN1157)[11]; these targeted polymorphic microsatellite loci located on 6 different chromosomes (Table 5). A PCR reaction of 10 μl was prepared containing 1 μl of 10 × AmpliTaq Gold Buffer (Applied Biosystems, USA), 1.0 μl of 25 mM MgCl2, 0.08 μl of 25 mM dNTPs, 0.1 μl of 10 μg/μl BSA, 0.05 μl of 5 U/μl AmpliTaq Gold enzyme (Applied Biosystems, USA), 0.24 μl of a 5'-fluorescent dye-labeled 20 μM forward primer (FAM, NED, PET or VIC), 0.24 ul of 20 μM reverse primer, 5.79 μl of distilled water, and 1.7 μl of DNA extract. PCR reactions were done under the following thermo-cycling condition: 95°C for 10 minutes followed by 50 cycles of each 95°C for 15 seconds, 55°C for 30 seconds and 72°C for 1 minute. The PCR products were visualized under ultraviolet light on a 2% agarose gel stained with ethidium bromide to verify amplification and the amplicons were then fractionated and sized on ABI3730 DNA Sequencer using GENEMAPPER v 4.0 (Applied Biosystems, USA). The PCR amplification and genotyping of each locus for each sample was done in triplicate. We included a positive snow leopards sample with each 3730 injection to ensure the alleles were size consistently and also ran positive and negative samples with each analysis.

**Table 5:** List of six microsatellite primer sequences used for genotyping for individual snow leopard identification.

**S. No**.	Primers	Sequence
**1**	PUN124-F	NED-5'-CCATTCCCTCCCTGTCTGTA-3'
	PUN124-R	5'-TGTCCTCAAACCATAGACAGTTTC-3'
**2**	PUN132-F	NED-5'-CGAAATGCAGTAATGTTAGTTTTACA-3'
	PUN132-R	5'-CACGGGTTCGTCTCTTTTG-3'
**3**	PUN229-F	VIC-5'-AGACAAACTGACAAGCTTAGAGG-3'
	PUN229-R	5'-TCATGTCTTTACATTCATTTCTTTTT-3'
**4**	PUN894-F	VIC-5'-CATGCCAGACTGCATTTGTT-3'
	PUN894-R	5'-CCCACACATGACAATCCTGTT-3'
**5**	PUN935-F	FAM-5'-GCTGCTGTGACCTTCTGTGA-3'
	PUN935-R	5'-CAGTGTTCCTGGTTTGCTCA-3'
**6**	PUN1157-F	FAM-5'-GAGAGTGCAGTCAGCCAGGT-3'
	PUN1157-R	5'-TGAAATTCAGCTGCTTCAACTC-3'

### Data Analysis

There were no independent population samples available of snow leopards from Nepal and we therefore estimated allele frequencies from the unique genotypes observed. The Probability of Identity for unrelated individuals (P_ID-unrelated_) and siblings (P_ID-siblings_) was estimated in GenAlex. Genetic diversity was estimated based on the mean number of alleles, observed heterozygosity and expected heterozygosity. We compared the distribution of snow leopards and the number of individuals across the two study sites. As our samples were not collected in a formal survey and we had small sample numbers, we could not estimate population size or density.

### Statement of ethical approval

This study was carried out with approval from the Department of National Parks and Wildlife Conservation, Ministry of Forests and Soil Conservation, the Government of Nepal (Reference no. 066/67 1548). The research used only non-invasively collected scat; hence there was no direct handling of the animals during our project.

## Abbreviations

CMDN: Center for Molecular Dynamics Nepal; DNA: Deoxyribonucleic acid; ID: Identification; KCA: Kangchanjunga Conservation Area; PCR: Polymerase Chain Reaction; SLIMS: Snow Leopard Information Management System; SPNP: Shey Phoksundo National Park; WWF-Nepal: World Wildlife Fund/Nepal.

## Competing interests

The authors declare that they have no competing interests.

## Authors' contributions

All authors have contributed to the design of the study, interpretation of data and read and approved the final manuscript. KT and RS from WWF/Nepal carried out all the field work, including sample collection. DBK and JEJ were involved in the molecular biology work, data analysis, and writing of the manuscript. MD helped in wildlife genetics research policy matters and logistics of field work.
